# Desempenho dos Índices de Gravidade na Predição de Complicações Pós-Operatórias de Revascularização Miocárdica

**DOI:** 10.36660/abc.20200515

**Published:** 2020-09-18

**Authors:** Alexandre de Matos Soeiro

**Affiliations:** 1 Instituto do Coração Universidade de São Paulo Unidade de Emergência São Paulo SP Brasil Instituto do Coração, Universidade de São Paulo - Unidade de Emergência,São Paulo, SP – Brasil; 2 Hospital BP Mirante Unidade Cardiológica Intensiva São Paulo SP Brasil Hospital BP Mirante - Unidade Cardiológica Intensiva, São Paulo, SP – Brasil

**Keywords:** Doenças Cardiovasculares/cirurgia, Revascularização Miocárdica, Procedimentos Cirúrgicos Cardíacos, Cuidados Pós-Operatórios, Escores da Disfunção Orgânica, Mortalidade

No Brasil não existem grandes estudos que relatam a experiência cirúrgica dos serviços de saúde associando à predição de eventos. Nesse contexto, o estudo “Desempenho dos índices de gravidade na predição de complicações pós-operatórias de revascularização miocárdica” traz uma informação relevante ao que se refere à literatura nacional. Esse estudo apresentado vem de maneira detalhada expor dados relevantes à ocorrência ou não de complicações perioperatórias em pacientes submetidos à cirurgia de revascularização miocárdica (CRM), correlacionando as mesmas com escores de predição de risco. Trata-se de tema extremamente relevante e que gera inúmeras incertezas frente ao paciente.^1^

A cirurgia cardíaca ainda representa um enorme campo no arsenal terapêutico dentro da cardiologia. Apesar de todos os avanços nas intervenções percutâneas coronarianas ou valvares, muitos casos permanecem ainda voltados à abordagem cirúrgica convencional. Hoje a cirurgia cardíaca convencional tem atuado cada vez mais com casos altamente complexos. Essa situação faz com que pacientes progressivamente de mais alto risco e complexidade anatômica coronariana sejam encaminhados à CRM. Dessa forma, predizer risco operatório adequadamente antes de um procedimento trata-se de uma etapa fundamental e obrigatória. Vale ressaltar que a maioria dos escores como *EuroScore* I e II ou *the Society of Thoracic Surgeons (STS) risk score* foram desenvolvidos para predizer mortalidade. *EuroScore* é um sistema de pontuação prognóstica desenvolvido na Europa para pacientes submetidos à cirurgia cardíaca. O que esse estudo nos traz é uma avaliação complementar e diferenciada de morbidade e disfunção orgânica em diferentes sistemas, como cardiovascular, neurológica, respiratória e renal.^1-5^

Estudo publicado em 2016 por Ad et al.,^6^ comparou em 11.788 pacientes durante 15 anos de cirurgias cardíacas à capacidade de predição de mortalidade entre os escores *EuroScore I, EuroScore II* e o *STS risk score*. Com mortalidade total de apenas 1,8% a área sob a curva encontrada foi respectivamente de 0,819, 0,844 e 0,846. Todos os escores provaram em larga casuística terem utilidade nessa população quando aplicados à prática clínica.^6^

Uma metanálise publicada também em 2016 incluiu 22 estudos que avaliaram o *EuroScore* e o *STS risk score* na predição de cirurgias valvares realizadas entre 2012 e 2015. O estudo concluiu mais uma vez que os escores eram similares na predição de mortalidade em 30 dias. No entanto, somente 3 dos estudos avaliados possuíam mais de 200 eventos documentados no seguimento, resultado esse considerado como fundamental pelos autores para predição adequada de eventos. Além disso, mais uma vez não foram avaliadas disfunções orgânicas imediatas do pós-operatório, ressaltando a importância desse tipo de dado no cenário atual.^7^

Outra metanálise recente com 145.492 pacientes incluídos submetidos à cirurgia cardíaca, analisou a performance do *EuroScore II* na predição de mortalidade. Na casuística estudada, a mortalidade real foi de 2,95%, tendo sido predita pelo *EuroScore II* de 3,3%. A área sob a curva na predição de morte foi de 0,792. Dessa forma, novamente o *EuroScore II* mostrou-se um bom preditor de eventos perioperatórios em cirurgia cardíaca.^8^

Apesar de toda a validação do *EuroScore* na predição de mortalidade, especificamente em relação à disfunções orgânicas sistêmicas, ele carece de avaliação. Já em relação aos outros escores, a utilização do *SOFA* foi avaliada em pós-operatório de cirurgia cardíaca, mostrando-se um bom índice novamente em avaliação de mortalidade.^9,10^ Estudo prospectivo com 1.058 pacientes em pós-operatório de cirurgia cardíaca avaliou a performance de diferentes escores em relação à mortalidade intra-hospitalar. A área sob a curva do escore *SOFA* foi de 0,929 para o valor médio e 0,927 para o valor máximo. Já o valor da área sob a curva para *EuroScore II* foi de 0,906.^10^

Por último, outro estudo avaliou em uma coorte de 36.632 pacientes os escores *SOFA, APACHE-II* e *SAPS-II* n predição de mortalidade em pós-operatório de cirurgia cardíaca. As áreas sob curva ROC foram de 0,809, 0,892 e 0,912, respectivamente. Nesse estudo, o escore *SOFA* mostrou o pior resultado comparativo, e mais uma vez não houve descrição específica de disfunções orgânicas individualmente.^11^

Dessa forma, a avaliação na população brasileira de escores nessa população após CRM torna-se importante. A capacidade de predizer disfunções orgânicas separadamente trata-se de algo pouco explorado e com relevância significativa agregando morbidade e tempo de internação.
