# Supplementary material for: Desempenho dos Índices de Gravidade na Predição de Complicações Pós-Operatórias de Revascularização Miocárdica
Source: Arq Bras Cardiol. 2020 Sep 18;115(3):460–1. [Article in Portuguese] doi: 10.36660/abc.20200515 (PMC9363087; doi:10.36660/abc.20200515)
Supplement: Translation

# Translation: Severity Index Performance in Predicting Postoperative Complications of Coronary Artery Bypass Grafting

**Authors:** Alexandre de Matos Soeiro

**Affiliations:** 1 Brazil Instituto do Coração, Universidade de São Paulo - Unidade de Emergência,São Paulo, SP – Brazil; 2 Brazil Hospital BP Mirante - Unidade Cardiológica Intensiva, São Paulo, SP – Brazil

**Keywords:** Cardiovascular Diseases/surgery, Myocardial Revascularization, Cardiac Surgical Procedures, Postoperative Car Organ Dysfunction Scores, Mortality

In Brazil, there are no large studies that reported the surgical experience of health services associated with event prediction. In this context, the study “Severity index performance in predicting postoperative complications of myocardial revascularization” provides relevant information regarding the national literature. This study shows, in a detailed manner, data relevant to the occurrence or not of perioperative complications in patients submitted to Coronary Artery Bypass Grafting (CABG) surgery, correlating them with risk prediction scores. This is an extremely relevant topic that generates countless doubts concerning the patient.^1^

Cardiac surgery still represents a huge field in the therapeutic arsenal of cardiology. Despite all the advances in percutaneous coronary or valvular interventions, many cases still remain focused on the conventional surgical approach. Currently, conventional cardiac surgery has been increasingly working with highly complex cases. This situation makes patients who are progressively at higher risk and who exhibit coronary anatomical complexity to be referred to CABG. Therefore, adequately predicting the operative risk before a procedure becomes a fundamental and mandatory step. It is worth mentioning that most scores such as EuroScore I and II or the Society of Thoracic Surgeons (STS) risk score were developed to predict mortality. The EuroScore is a prognostic scoring system developed in Europe for patients undergoing cardiac surgery. What this study brings us is a complementary and differentiated assessment of morbidity and organ dysfunction in different systems, such as the cardiovascular, neurological, respiratory and renal systems.^1-5^

A study published in 2016 by Ad et al.^6^ compared 11,788 patients during a 15-year period of cardiac surgeries regarding the ability to predict mortality between the EuroScore I, EuroScore II scores and the STS risk score. With a total mortality rate of only 1.8%, the area under the curve found was 0.819, 0.844 and 0.846, respectively. All scores have shown to be largely useful in this population, when applied to clinical practice.^6^

A meta-analysis also published in 2016 included 22 studies that assessed the EuroScore and STS risk score regarding the prediction of valvular surgeries performed between 2012 and 2015. The study concluded once again that the scores were similar in the 30-day mortality prediction. However, only 3 of the evaluated studies had more than 200 events documented during the follow-up, a result considered essential by the authors for adequate event prediction. Additionally, once again immediate postoperative organ dysfunction was not evaluated, emphasizing the importance of this type of information in the current scenario.^7^

Another recent meta-analysis with 145,492 patients undergoing cardiac surgery, analyzed the performance of EuroScore II in predicting mortality. In the studied sample, the actual mortality rate was 2.95%, having been predicted by EuroScore II as being 3.3%. The area under the curve for the mortality prediction was 0.792. Thus, EuroScore II again showed to be a good predictor of perioperative events in cardiac surgery.^8^

Despite all the EuroScore validation in predicting mortality, specifically in relation to systemic organ dysfunction, it lacks evaluation. In relation to the other scores, the use of SOFA was evaluated in the postoperative period of cardiac surgery, showing again a good index in assessing mortality.^9,10^ A prospective study with 1,058 patients in the postoperative period of cardiac surgery evaluated the performance of different scores in relation to in-hospital mortality. The area under the curve of the SOFA score was 0.929 for the mean value and 0.927 for the maximum value. The value of the area under the curve for EuroScore II was 0.906.^10^

Finally, another study evaluated SOFA, APACHE-II and SAPS-II scores in a cohort of 36,632 patients for the prediction of mortality in postoperative cardiac surgery. The areas under the ROC curve were 0.809, 0.892 and 0.912, respectively. In this study, the SOFA score showed the worst comparative result, and again there was no specific description of individual organ dysfunctions.^11^

Thus, the evaluation in the Brazilian population of scores in this group of patients after CABG becomes important. The ability to predict organ dysfunctions individually is something that has been little explored and is of significant relevance, aggregating morbidity and hospital length of stay.
